# Reconfigurable Single-Layer Graphene Radio Frequency Antenna Device Capable of Changing Resonant Frequency

**DOI:** 10.3390/nano13071203

**Published:** 2023-03-28

**Authors:** Hyeon Jun Hwang, So-Young Kim, Sang Kyung Lee, Byoung Hun Lee

**Affiliations:** 1 Center for Semiconductor Technology Convergence, Department of Electrical Engineering, Pohang University of Science and Technology, Cheongam-ro 77, Nam-gu, Pohang 37673, Gyeongbuk, Republic of Korea; hhjune@postech.ac.kr (H.J.H.); una0918@gmail.com (S.-Y.K.); 2 Alphagraphene Inc. 77, Cheongam-ro, Nam-gu, Pohang 37673, Gyeongbuk, Republic of Korea; alphagraphene.korea@gmail.com

**Keywords:** reconfigurable passive device, resonant frequency, graphene, capacitance change, frequency shift

## Abstract

A reconfigurable passive device that can manipulate its resonant frequency by controlling its quantum capacitance value without requiring complicated equipment has been experimentally investigated by modifying the Fermi level of large-area graphene using an external electric field. When the total capacitance change, caused by the gate bias in the passive graphene device, was increased to 60% compared to the initial state, a 6% shift in the resonant frequency could be achieved. While the signal characteristics of the graphene antenna are somewhat inferior compared to the conventional metal antenna, simplifying the device structure allowed reconfigurable characteristics to be implemented by using only the gate bias change.

## 1. Introduction

Graphene is a monolayer material but acts as an interface for bonding with other materials. Consequently, the primary area of study regarding the constant voltage condition has been the analysis of the resistance change of a device due to the interface charging phenomenon. For radio frequency (RF) applications, the inductance and capacitance of the device greatly affect the operating characteristics. Moreover, unlike metal, most signals applied from the outside would be transmitted, rather than absorbed or reflected, owing to graphene’s thinness (theoretically ~0.3 nm) and possibly being due to the frequency dependence of skin depth. This suggests that the material with a thickness in nm is more reactive in the THz region [1,2,3].

Early studies regarding the RF characteristics of graphene devices primarily focused on applied research, using a high cutoff frequency and the frequency-doubling effect in field-effect transistor (FET)-type active devices [4,5,6,7,8]. Graphene FET (GFET)’s ground–signal–ground (GSG) pad structure, used for rapid signal propagation and amplification, was used for studying frequency ranges in the THz and optical domains. For these studies, exfoliated graphene or small-patterned chemical vapor deposition (CVD) graphene was used [4,9]. Passive RF devices have been investigated using graphene [10,11,12,13,14,15,16,17,18,19,20,21,22,23]. Most of the reports on passive devices using graphene, such as antennas and RF filters, analyzed them using theoretical approaches [20,21] because the size of graphene required to fabricate the passive devices was not available. For example, the size of an antenna resonating at 1 GHz requires a ~30 cm wire. For practical applications, GHz signals can be transmitted and received using shorter antennas by utilizing the multiple resonance phenomena and signal amplification techniques [11]. The drawback of this approach is the higher energy consumption required for signal processing. If a graphene antenna is too small compared to the wavelength, it cannot distinguish between ambient noise and signals. Thus, large-scale, high-quality graphene with an area of only a few millimeters is necessary for RF applications.

In this work, two dipole antennas integrated on the same plane were fabricated using a four-inch wafer-scale graphene sheet. When graphene was used as an antenna, a 2% level of resonance-frequency modulation was observed, according to the 10% change in capacitance. In addition, when the graphene was used as a ground plane, a 6% level of resonance-frequency modulation was successfully achieved due to the 60% capacitance change achieved via the quantum capacitance of the graphene sheet. For reconfigurable passive devices requiring resonant frequency control [24,25], a resonant frequency change rate corresponding to 2.5~9.6% of the capacitance change rate in the passive graphene device was possible.

## 2. Methodology: Structure of the Passive Graphene Device and Fabrication

To create the graphene-antenna device, a 50 nm oxide trench pattern was formed on a SiO_2_ (300 nm)/Si substrate using a reactive ion-etching process. Subsequently, a 50 nm Pt electrode was deposited inside the trench by using an E-beam evaporation system to form a buried-gate electrode with significant advantages over the other structures in terms of the uniformity of the electric field applied to the channel region. To achieve planarization of the gate electrode, using chemical and mechanical polishing processes, 20 nm of Al_2_O_3_ was deposited using an atomic layer deposition (ALD) process and a 300 °C vacuum-annealing process for 1 h. Subsequently, the 4-inch-scale CVD graphene sheets (Alphagraphene Inc., Pohang, Republic of Korea) were transferred using a vacuum-transfer method to ensure a stable and consistent device operation, considering that this method can reduce impurities, such as oxygen and moisture, between the graphene and the substrate [26]. To prevent PR residues from appearing on the graphene channel, which may occur when patterning the graphene channel using the O_2_ plasma, the graphene area was patterned via a photolithography process using a metal hard mask (Au). Finally, the top electrode (Au) was formed using an E-beam evaporation process and wet etching.

**Figure 1:**
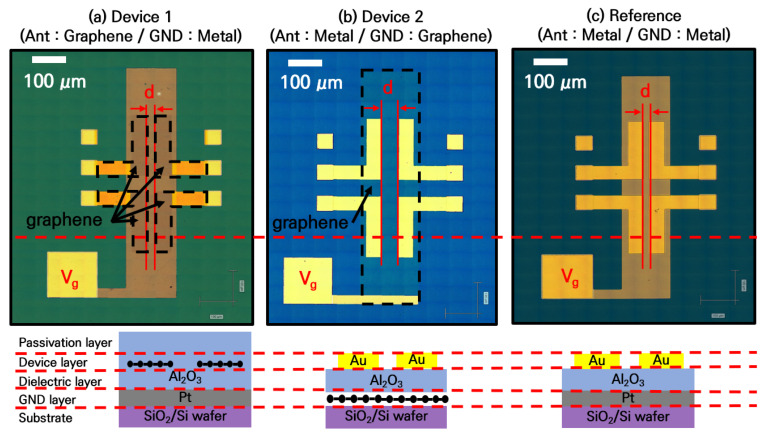
Optical images of the RF passive devices. Two dipole antennas are integrated on the same plane, and the ground plane is used as a gate to control the graphene Fermi level. (**a**) Device 1: has an antenna consisting of graphene patterns and ground metal. (**b**) Device 2: Graphene is used as the ground plane and the antenna is made of a metal film to increase the signal transmission rate of the device and induce a quantum capacitance change in graphene. (**c**) Reference device fabricated with Au patterns.

A passive device with a transmitter and receiver of two basic dipole antennas integrated on the same plane was fabricated to study the reconfigurable passive device using graphene. Device 1 consisted of 100 μm pitch ground–signal–ground (GSG) pads, as shown in Figure 1a, with a graphene antenna width of 50 μm and length of 450 μm. The thickness of the Al_2_O_3_ gate dielectric was 20 nm (dielectric constant = 6). The antenna itself was simply designed to operate at approximately 660 GHz, with resonance at 10–20 GHz. The ground plane was made of metal and platinum (Pt) to control the Fermi level of the graphene. The spacing between the transmitter and the receiver was alternated between 10 and 150 μm to observe the changes in signal-transmission characteristics. The Fermi level of the graphene could be controlled using the bias applied to the V_g_ contact shown in Figure 1a. Device 2, shown in Figure 1b, was a variation of Device 1 in which the ground plane was replaced with graphene. As the device capacitance was determined by the overlapping region of the metal and graphene, the total capacitance was expected to be similar to that of Device 1. As a reference, a similar device made of a metal (Au) film was fabricated, as shown in Figure 1c.

Its characteristics in the RF domain were analyzed using a network analyzer (Agilent 8510C) and a two-port probe system in the 1–40 GHz range. The S-parameters, reflections (S11, S22), and transmissions (S12, S21) were measured using a network analyzer. A constant voltage applied to the ground plane modulated the Fermi level of the graphene. Then, the S-parameters were measured as a function of the Fermi level of the graphene. First, we observed the appearance of signal reflections and transmissions when the spacing between the three antennas physically changed. The Fermi level of the graphene for the graphene antenna was assumed to be at the Dirac point. The observation of the Dirac point, based on the doping condition of graphene devices, can generally be obtained by measuring the I_d_–V_g_ (drain current–gate voltage). In a passive device, such as Device 1, a DC current does not flow. Hence, the gate voltage value for accurate Dirac point setting was unknown during the RF measurements. Moreover, Dirac point analysis in graphene devices is possible using C–V measurements instead of I_d_–V_g_ measurements. However, reconfiguring the four electrodes into one electrode for correct C–V measurements would be necessary, as two dipole antennas were patterned with graphene during the formation of Device 1. A capacitance measurement was carried out using the top electrode and buried electrode on the same substrate of the antenna device. Consequently, the Fermi level change in the graphene at the S-parameter measurement was set to a point where the Dirac point appeared at a gate voltage of 0 V. In this case, the estimated carrier density existing at the charge neutrality point was 3.1 × 10^11^cm^−2^. This value is very important because the quantum capacitance of graphene is generally more clearly observed when it has a lower carrier density than the classical capacitance of the device, and this is the ultimate reason for the tunability of the resonance frequency in these devices [27].

## 3. Results: RF Characteristics of the Reconfigurable Passive Graphene Device

**Figure 2:**
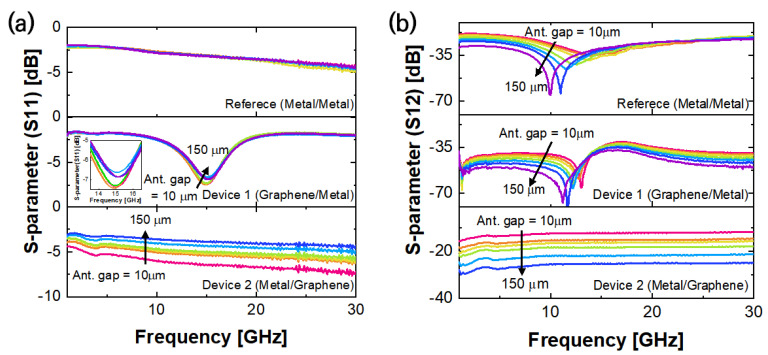
S-parameter measurement results based on the distance between the three antennas. (**a**) Reflectance (S11) and resonant frequency increase as the gap increases. (**b**) Transmittance (S12) and resonant frequency decrease as the gap increases.

Figure 2 shows the S-parameter values that were measured while alternating the antenna spacing between 10μm and 150μm. In the case of S11, the metal–metal structure showed a gradual decrease as the frequency increased. The graphene–metal structure showed a resonant peak at ~18 GHz. The inset of Figure 2a for the graphene–metal structure shows a gradual peak shift towards a higher frequency with wider gaps. On the other hand, the metal–graphene structure showed similar behavior as that of the metal–metal reference. As the distance between the antennas in Device 1 increased, the resonant frequency shifted towards a lower frequency and the overall transmittance decreased. The characteristics of the reference passive device were also measured under the same conditions. When the distance between the antennas was small, the resonance phenomenon was not clear, unlike with the graphene RF device. However, when the distance between the antennas was more than 100 μm, the resonant frequency became apparent. The reference device exhibited a higher transmittance value than Device 1 (by approximately 15 dB), but its reflectance value (S11) did not exhibit any resonance phenomena.

In the case of S12, as shown in Figure 2b, as the antenna spacing increased, the resonant frequency shifted to lower frequencies and its value decreased, i.e., as the antenna spacing increased, signals of lower frequencies were transmitted better, while the signals of higher frequencies were mostly reflected at the input port. Consequently, as the distance between the antennas widened, the size of the ground plane increased, i.e., the capacitance of the entire device increased, and the high-frequency components were filtered.

The S-parameter measurements of the RF operating characteristics of Device 2 did not exhibit resonance at a specific frequency as they did in Device 1 or in the reference device. For Device 2, analysis was conducted based on the characteristic changes at 4.5 GHz. When the distance between the metal antennas was increased in Device 2, a change in the S-parameter value over the entire frequency range, rather than a resonant frequency shift, was observed. In particular, S12 or S21 showed signal transmission attenuations of more than 15 dB when the distance was increased. Simultaneously, in S11 or S22, the reflection amount increased as the distance between the antennas increased.

Next, the RF characteristics were investigated while applying the external bias to the graphene to modulate the Fermi level of the graphene. Using a gate electrode as a ground plane, measurements were made at −5, −3, −1, 0, 1, 3, and 5 V; different polarities were applied to observe the changes in the graphene carrier types in the graphene layer.

**Figure 3:**
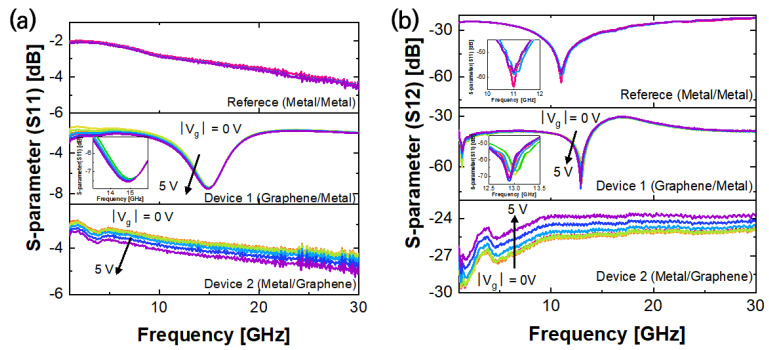
S-parameter measurement results with gate bias control. (**a**) Reflectance (S11) and (**b**) transmittance (S12).

As resonance was most apparent when the antenna spacing was at 100 μm, the characteristics based on the gate bias change were observed in the device at that spacing. The results, based on changing the gate bias, did not differ much compared to those obtained when the antenna spacing was changed, as shown in Figure 3. As the value of the gate bias increased, the reflection decreased and the resonant frequency shifted to a lower frequency band. However, since there was no change to S11 over a frequency range of 15.5 GHz, it is difficult to appreciate that the resonant frequency shift is similar, i.e., the increase in the resonance bandwidth could be predicted. In S12, as shown in the Figure 3b inset, a parallel shift in the resonant frequency was observed, unlike in S11. As the V_g_ increased, the resonant frequency shifted towards the lower frequencies. The frequency shift range was approximately 150 MHz. The change in characteristics due to the V_g_ was shown in that the degree of the shifting of the resonant frequency was proportional to the absolute value, regardless of the polarity of the V_g_, i.e., when the Fermi level of graphene changed via the gate bias, the carrier type and carrier concentration changed based on this value and the polarity of the gate bias. However, as seen from the I_d_–V_g_ measurements of the graphene device, the same conductance could be seen even if the absolute value of the gate bias was the same, regardless of the carrier type. Therefore, as shown in Figure 3, the change in resonant frequency in the passive graphene device was independent of the graphene carrier type but was related to the carrier concentration. As the voltage applied to regulate the graphene Fermi level was constant, the charge trap at the graphene interface in the device could be sensitive to certain carrier types and affect the device’s performance.

Although Device 1 and the graphene RF device exhibited similar resonance characteristics to those of the reference passive device under its operating conditions, their transmittance values were low and their reflectance values were very high, possibly due to the significantly high contact resistance between the graphene and the graphene–metal contact. Therefore, improving the structure of the passive device would be necessary to secure its basic performance while facilitating the movement of resonant frequency by controlling the quantum capacitance of the graphene. The changes in the resonant frequency and measured S-parameter values due to changes in the Fermi levels of graphene, used as a ground plane by the gate bias, were more apparent than those in Device 1. As the V_g_ increased, the signal transmission improved by 3 dB and the reflectance reduced by 1 dB, as shown in Figure 3.

**Figure A1:**
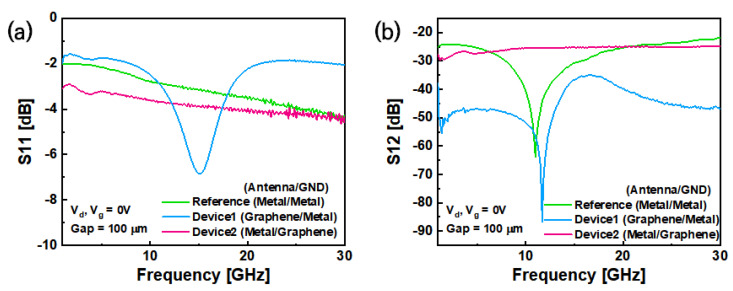
The measured values of Device 1, Device 2, and the reference device, under the same conditions, were compared to analyze the cause of different signal (**a**) reflection and (**b**) transmission characteristics.

Under the same conditions, the measured values of Device 1, Device 2, and the reference device were compared to analyze the cause of different signal transmissions and reflection characteristics, as shown in Figure A1. For S11, the response of the reference device and Device 2 were similar when using metal antennas. For S12, the reference device and Device 1 exhibited similar responses when both used metal as the ground plane. Consequently, the reflection value of the device significantly influenced the impedance value of the antenna, and the transmission value significantly influenced the condition of the ground plane. The graphene antennas exhibited resonance at 15 GHz, but they have a very high reflection value in the remaining frequency range.

Conversely, in the case of signal transmission (S12, S21), the impedance value of the passive device itself and the space for transmitting the RF signal were important [25]. The RF signal was not transmitted through the surface of the metal, but rather through the space between the metal and ground plane such that a specific energy wavelength was emitted. Therefore, the state of the ground plane was an important aspect of the operating characteristics of the passive device. The problem arises from the graphene being used as an electrode, where the energy absorption rate of the electric field is low. However, graphene used as an electrode (ground) in actual passive devices has not been able to fulfill the role of a metal film. The electric field associated with graphene’s Fermi level control is the same energy corresponding to this bias frequency. Therefore, examining how RF signals in the GHz range can be applied to graphene from a different perspective is necessary. Previous research has found that if graphene is placed in a floating state without any electrical contact, approximately 20% of the RF signal is absorbed and the remainder is reflected or transmitted [23]. Therefore, independent of the Fermi level control using a DC bias, 20% of the RF signal energy can be expected to be absorbed from the graphene ground plane, and the remaining part should be reflected, i.e., the RF signal will not be radiated properly between the antennas and the ground plane. In Device 1, this problem was eliminated as the RF signals were emitted from the graphene antennas, and the flow of the surface current was formed based on the graphene pattern.

**Figure 4:**
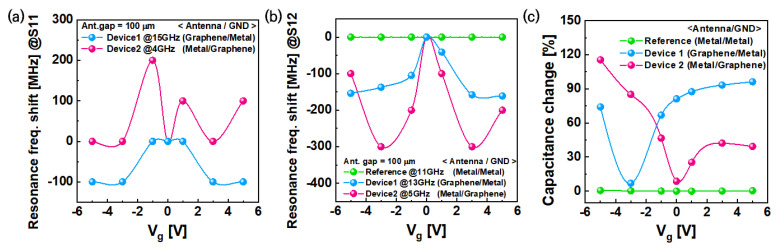
(**a**) For S11, the resonant frequency shift in reflectance is shown to be opposite when using graphene as an antenna and as a ground plane. As the graphene Fermi level increases in Device 1, the signal reflection in the low-frequency band increases. When the graphene Fermi level increases in Device 2, the signal reflection in the high-frequency band increases. Conversely, (**b**) for S12, all of the resonant frequency shifts were towards the lower frequency, exhibiting approximately twice the level of change in Device 1 compared to Device 2. (**c**) The capacitance variation according to the gate voltage change is plotted. When graphene is applied to the device, a capacitance change of approximately 120% is observed.

Consequently, the degree of resonant frequency variation regarding the reflectance and transmittance due to the V_g_ was compared, as shown in Figure 4a,b. For the reference passive device, no resonant frequency change due to the V_g_ could be observed. However, a change in resonant frequency could be seen when the graphene was used. For S11, when Device 1 was exposed to a larger V_g_ value, an approximately 100 MHz shift towards the lower frequency was observed. However, for Device 2, the resonant frequency increased by approximately 200 MHz. The analysis results of Figure 4 show that the impedance of the antenna was affected more by a change in the S11 characteristics. An increase in the resonant frequency bandwidth indicated an improvement in signal transmission in the 15 GHz band. In Device 2, the resonant frequency shift and reflection decrease in the 4 GHz band could be attributed to an increase in carrier concentration caused by changes in Fermi levels. This resulted in improved signal transmission through the graphene ground plane. For S12, the shift width of the resonant frequency was more than that of S11, as shown in Figure 4. For both Device 1 and 2, the resonant frequency changed due to the V_g_ occurring approximately 1.5 times more, i.e., Device 1 and Device 2 exhibited a resonant frequency change of approximately 200 MHz and 300 MHz, respectively.

**Figure A2:**
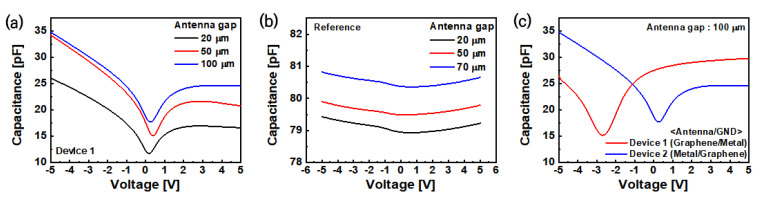
Comparison of capacitance variation of passive devices. (**a**) In Device 1, the capacitance value increases as the distance between the antennas increases. (**b**) For the reference device, there is no capacitance change due to quantum capacitance because graphene is not applied to the device. (**c**) Comparison of capacitance values of Device 1 and Device 2.

Increasing the carrier concentration of graphene in passive devices improved the RF signal transmission, and the quantum capacitance changes induced a change in the resonant frequency. The capacitance value of each passive device was measured to examine the correlation between the S-parameter characteristics and the capacitance changes in the passive device, as shown in Figure 4c. For Device 2 (Figure A2a), where graphene was used as the ground plane, a capacitance change of approximately 15 pF was measured due to the graphene quantum capacitance changes. Although the capacitance of the reference passive device (Figure A2b) was higher than that of Devices 1 and 2, the capacitance variation by the Vg was approximately 0.5 pF, which is approximately 30 times lower. Figure A2c compares the capacitance measurement results of Device 1, which used graphene as the antenna, and Device 2, which used graphene as the ground plane. The Dirac point not appearing near 0 V implies that the graphene was doped. As the Dirac point of Device 1 appears near −2.5 V, the graphene could be considered to be n-type. Since the Dirac point of Device 2 was measured by directly applying Vg to the graphene, the Fermi level shift was observed in the direction opposite to the graphene Fermi level shift in Device 1, i.e., as the Fermi level moved in the positive (p-type) voltage direction in Device 2, it mirrored the capacitance measurement value of Device 1. Figure 4c shows the rate of capacitance change. Owing to the changes in the quantum capacitance of the graphene, the capacitance value changed by approximately 120% as the Vg increased.

## 4. Discussion

**Figure 5:**
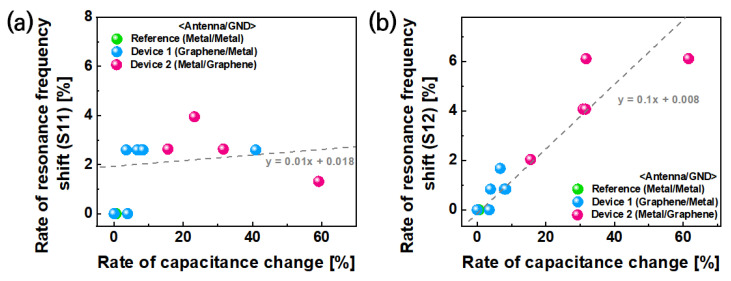
(**a**) Analysis of the relation between reflectance resonant frequency shift and graphene Fermi level change. (**b**) Analysis of the relation between transmittance resonant frequency shift and graphene Fermi level change.

In this experiment, we changed the resonant frequency in a passive device using graphene. The relationship between the rate of resonant frequency shift and the rate of capacitance change was investigated based on V_g_ = 0 V. As shown in Figure 5, 6% of the rate of transmittance resonant frequency shifted in correspondence with the rate of capacitance change that could be observed, regardless of the applied structure of graphene in the passive device. Additionally, the reflectance S-parameter value of the graphene RF devices changed minimally when compared with the reference device, and the rate of reflectance resonant frequency showed a 2% level shift, regardless of changes in the carrier concentration of graphene based on gate bias. Therefore, we can conclude that graphene RF devices can be reconfigured by gate bias without energy loss. When the total capacitance change of the passive device was 60% due to the graphene Fermi level change, a resonant frequency shift corresponding to 6% of the original resonant frequency value occurred.

## 5. Conclusions

To design a reconfigurable passive device using graphene, we fabricated integrated passive devices with two dipole antennas and analyzed their RF characteristics, aiming to analyze the use of graphene in passive devices and the amount of resonant frequency change possible based on the changes in V_g_. When graphene was used as an antenna, characteristics similar to those of a passive device using a conventional metal film were observed, suggesting that graphene could be applied as a passive device based on the patterning. However, when a graphene ground plane was used, small-signal RF transmission was compromised and an accurate passive device operation could not be performed. Nonetheless, changes in quantum capacitance and carrier concentration due to graphene Fermi level changes affected the resonant frequency control and signal propagation. For reconfigurable passive devices requiring resonant frequency control, a resonant frequency change rate corresponding to 2.5~9.6% of the capacitance change rate in the passive graphene device was possible. Although these changes are insufficient for the practical application of graphene in reconfigurable passive devices, they indicate the potential to explore and positively contribute to their development.

## Data Availability

Not applicable.
